# Twin epidemics: the effects of HIV and systolic blood pressure on mortality risk in rural South Africa, 2010-2019

**DOI:** 10.1186/s12889-022-12791-z

**Published:** 2022-02-24

**Authors:** Brian Houle, Chodziwadziwa W Kabudula, Andrea M Tilstra, Sanyu A Mojola, Enid Schatz, Samuel J Clark, Nicole Angotti, F Xavier Gómez-Olivé, Jane Menken

**Affiliations:** 1 School of Demography, The Australian National University, Canberra, Australia; 2 MRC/Wits Rural Public Health and Health Transitions Research Unit (Agincourt), Faculty of Health Sciences, School of Public Health, University of the Witwatersrand, Johannesburg, South Africa; 3 Nuffield College, University of Oxford, Oxford, UK; 4 Institute of Behavioral Science and Department of Sociology, University of Colorado Boulder, Boulder, USA; 5 Leverhulme Centre for Demographic Science, Department of Sociology, University of Oxford, Oxford, UK; 6 Department of Sociology, School of Public and International Affairs, and Office of Population Research, Princeton University, Princeton, USA; 7 Department of Public Health, University of Missouri, Columbia, USA; 8 Department of Sociology, The Ohio State University, Columbus, USA; 9 Department of Sociology, American University, Washington, USA

**Keywords:** Mortality, Rural, South Africa, HIV, Hypertension, Blood pressure

## Abstract

**Background:**

Sub-Saharan African settings are experiencing dual epidemics of HIV and hypertension. We investigate effects of each condition on mortality and examine whether HIV and hypertension interact in determining mortality.

**Methods:**

Data come from the 2010 Ha Nakekela population-based survey of individuals ages 40 and older (1,802 women; 1,107 men) nested in the Agincourt Health and socio-Demographic Surveillance System in rural South Africa, which provides mortality follow-up from population surveillance until mid-2019. Using discrete-time event history models stratified by sex, we assessed differential mortality risks according to baseline measures of HIV infection, HIV-1 RNA viral load, and systolic blood pressure.

**Results:**

During the 8-year follow-up period, mortality was high (477 deaths). Survey weighted estimates are that 37% of men (mortality rate 987.53/100,000, 95% CI: 986.26 to 988.79) and 25% of women (mortality rate 937.28/100,000, 95% CI: 899.7 to 974.88) died. Over a quarter of participants were living with HIV (PLWH) at baseline, over 50% of whom had unsuppressed viral loads. The share of the population with a systolic blood pressure of 140mm Hg or higher increased from 24% at ages 40-59 to 50% at ages 75-plus and was generally higher for those not living with HIV compared to PLWH. Men and women with unsuppressed viral load had elevated mortality risks (men: adjusted odds ratio (aOR) 3.23, 95% CI: 2.21 to 4.71, women: aOR 2.05, 95% CI: 1.27 to 3.30). There was a weak, non-linear relationship between systolic blood pressure and higher mortality risk. We found no significant interaction between systolic blood pressure and HIV status for either men or women (*p*>0.05).

**Conclusions:**

Our results indicate that HIV and elevated blood pressure are acting as separate, non-interacting epidemics affecting high proportions of the older adult population.

PLWH with unsuppressed viral load were at higher mortality risk compared to those uninfected. Systolic blood pressure was a mortality risk factor independent of HIV status. As antiretroviral therapy becomes more widespread, further longitudinal follow-up is needed to understand how the dynamics of increased longevity and multimorbidity among people living with both HIV and high blood pressure, as well as the emergence of COVID-19, may alter these patterns.

**Supplementary Information:**

The online version contains supplementary material available at 10.1186/s12889-022-12791-z.

## Background

South Africa is undergoing complex demographic and epidemiological transitions, with intersecting epidemics of HIV and hypertension. The country has one of the largest and fastest growing older populations in sub-Saharan Africa [1]. Globally, South Africa has one of the highest levels of HIV prevalence and the largest antiretroviral therapy (ART) program [2]. The success of widespread ART rollout has led to population-level declines in mortality [3, 4], resulting in the aging of the epidemic as people living with HIV (PLWH) who are on ART live longer [5]. However, gaps remain in the HIV treatment cascade – amongst adults aged 40-plus in rural South Africa, 63% of those screened HIV-positive were on ART and, of those, 72% had viral suppression [6]. The burden of noncommunicable diseases (NCD) is also high [7], particularly the prevalence of hypertension [8, 9]. This is complicated by low awareness among those with a hypertensive condition: an estimated 38-64% of hypertensive people were aware of their status and only 8-23% had controlled blood pressure [9–11].

With widespread availability of ART in sub-Saharan Africa and resulting survival gains, it is critical to understand the evolving morbidity and mortality patterns of PLWH. Studies in high income countries have shown high levels of cardiovascular risk factors and events in PLWH [12–14], and mixed evidence on shifts in causes of death from AIDS to NCDs [15, 16]. Limited evidence from sub-Saharan Africa indicates persistent HIV-related hospitalizations and mortality due to AIDS despite being on ART [17–20].

It is unclear if HIV infection and ART interact with hypertension to increase mortality risk. Studies in Kenya and Haiti restricted to PLWH found associations between blood pressure and elevated mortality [21, 22]. However, a U.S. study of veterans living with HIV matched to uninfected veterans in care found that HIV and blood pressure were independently associated with mortality [23].

We use a population-based cross-sectional survey (ages 40-plus) and eight-year longitudinal mortality follow-up data from rural South Africa to examine associations between HIV status and systolic blood pressure and differential mortality risk.

## Methods

### Setting and data

Our data come from the 2010-2011 Ha Nakekela HIV and NCD Study and the Agincourt Health and socio-Demographic Surveillance System (Agincourt HDSS), situated in the rural northeast of South Africa. The Agincourt HDSS has collected longitudinal, population-level data annually on all vital events and socio-economic information since 1992 [24]. In 2011, the surveillance population comprised approximately 90,000 people residing in 27 villages. For the Ha Nakekela study, we randomly selected a sex-age stratified sample of 7,662 individuals using the 2009 Agincourt HDSS census round as the sampling frame, including an oversample of older adults [25, 26]. Inclusion criteria were men and women aged 15 and older who were permanent residents in the Agincourt HDSS study area based on information from the 2009 census round. A total of 4,362 individuals were eligible, consented to be interviewed and tested for HIV [26]. Of these, 3,024 were aged 40-plus. Data collected included blood tests and dried blood spots, anthropometric and blood pressure measurements, and a sexual behavior and chronic disease risk factor questionnaire. Follow-up information on mortality came from annual census update rounds of the Agincourt HDSS through mid-2019.

### Key measures

#### HIV status [25]

Dried blood spots were tested using screening assay Vironostika Uniform 11 (Biomeriuex, France). Positive results were retested using the SD Bioline HIV ELISA test (SD; Standard Diagnostics Inc., Korea). If the two tests were inconsistent, a third assay was conducted (Elecys, Roche, USA) to determine the final result.

#### Blood pressure [8]

Blood pressure was measured three times using a Boso BP instrument [27–29]. We used the average of the second and third measurements. Current use of antihypertensive medications was based on self-report.

### Analyses

We restricted this analysis to the 3,024 respondents aged 40-plus and, further, to those with complete covariate information, resulting in a final analytic sample of 2,909 adults: 1,802 women and 1,107 men. We restricted to those aged 40-plus as the risk of dying from systolic blood pressure-related diseases at younger ages was too low for systematic analysis. Baseline covariates included age, nationality (South African or other), marital status (single, married/cohabiting, widowed/divorced), years of education (0-3 years, 4-8 years, 8+ years), employment status (1/0), and household wealth tertile based on a household asset index [30]).

The primary covariates were HIV and systolic blood pressure. We categorized HIV as a combination of HIV diagnosis and viral load: (1) HIV negative; (2) HIV positive, viral load suppressed; and (3) HIV positive, viral load unsuppressed. Respondents were classified as suppressed if their viral load was less than 400 copies/mL and unsuppressed if their viral load was above 400 copies/mL [31]. In supplementary analyses, we assessed the sensitivity of our analysis to this threshold by considering a second HIV status variable that classified respondents as suppressed if their viral load was less than 1,000 copies/mL and unsuppressed if their viral load was at least 1,000 copies/mL [32]. We tested both systolic blood pressure and diastolic blood pressure. Since results were similar, we focus on systolic blood pressure but show diastolic blood pressure results in Additional File 1.

We structured the data into a person-year format, and used sex-stratified discrete-time models to characterize the risk of dying [33], adjusting for sample weighting and survey nonresponse [34]. The weights are a product of the inverse probability sample selection weights and inverse probability weights to adjust for nonresponse based on covariates from the 2009 census. Our discrete-time model is similar to the Cox proportional hazards approach, but has a number of advantages, including: greater flexibility in modelling temporal relationships and interactions; and results in the form of predicted probabilities which can be interpreted independently. Respondents were followed until either July 2019, their death, or censoring (out-migration or loss-to-follow-up). To identify potential non-linear relationships between systolic blood pressure and mortality, we modelled mortality as a quadratic function of systolic blood pressure. Tests modelling systolic blood pressure as a restricted cubic spline showed similar results. We also adjusted for self-reported use of medications to lower blood pressure. We tested for interactions between age and HIV status, age and systolic blood pressure, and systolic blood pressure and HIV status using nested likelihood ratio tests.

## Results

**Fig. 1:**
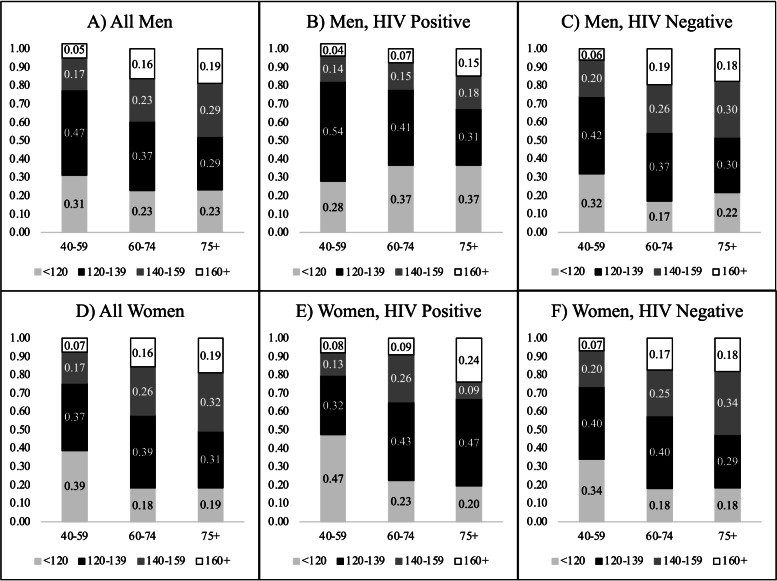
Age-specific distributions of systolic blood pressure categories by sex and HIV status, Agincourt, South Africa, 2010-11. Proportions are survey weighted

Table 1 presents descriptive results for the survey participants aged 40-plus. Over a quarter of men and women were living with HIV, over half of whom had unsuppressed viral loads in 2010-11. Figure 1 shows that the share of the population with a systolic blood pressure of 140mm Hg or higher significantly increased for men from 22% at ages 40-59 to 48% at ages 75-plus (*p*<0.001) and for women from 25% to 51% (*p*<0.001). The proportion with a systolic blood pressure of 140mm Hg or more was generally higher for those not living with HIV compared to PLWH (*p*<0.001 for both men and women), particularly at older ages. Mortality during the eight years of follow-up was high (477 deaths): survey weighted estimates are that 37% of men (mortality rate 987.53 per 100,000, 95% CI: 986.26 to 988.79) and 25% of women (mortality rate 937.28 per 100,000, 95% CI: 899.7 to 974.88) died.

**Table 1:** Descriptive statistics and tests of differences between men and women for the estimation sample, Agincourt, South Africa, 2010-11

	**Overall**	**Men**	**Women**	***P*-value**
	**Percent (%)**	**Percent (%)**	**Percent (%)**	**Difference**
Age Group				0.068
40-59	51.3	48.8	52.5	
60-74	24.7	27.8	23.3	
75+	24.0	23.4	24.3	
Missing (N)	0	0	0	
HIV Status				0.003
HIV Negative	73.0	73.0	73.0	
HIV Positive Suppressed^a^	11.5	8.6	12.8	
HIV Positive, Unsuppressed^b^	15.5	18.4	14.2	
Missing (N)	226	117	109	
Citizenship				0.007
South African	71.6	75.3	69.8	
Other	28.4	24.7	30.2	
Missing (N)	0	0	0	
Marital Status				<0.001
Single	15.1	18.1	13.7	
Married/cohabiting	46.7	60.1	40.3	
Widowed/divorced	38.2	21.7	46.1	
Missing (N)	3	1	2	
Education Level				<0.001
None/very low (≤3 yrs)	46.8	40.8	49.6	
Primary (4-8 yrs)	25.3	28.6	23.7	
Secondary school or higher (>8 yrs)	28.0	30.7	26.7	
Missing (N)	103	36	67	
Working Status				<0.001
Not working	70.8	62.7	74.6	
Working	29.2	37.3	25.4	
Missing (N)	173	61	112	
Household Wealth Tertile				0.403
1st (lowest wealth)	34.1	32.5	34.9	
2^nd^	21.3	20.9	21.5	
3^rd^	44.6	46.6	43.6	
Missing (N)	11	5	6	
	**Mean**	**Mean**	**Mean**	
Systolic Blood Pressure	133.80	133.6	133.9	0.789
Missing (N)	44	23	21	
Systolic Blood Pressure by HIV Status				
HIV Negative	136.23	136.3	136.2	0.931
HIV Positive, Suppressed^a^	126.24	125.6	126.5	0.780
HIV Positive, Unsuppressed^b^	128.23	128.8	127.9	0.681
Missing (N) on both SBP and HIV	35	21	14	
Died during 8-year follow-up	28.5	36.9	24.5	<0.001
*N*	2,909	1,107	1,802	

Data come from Ha Nakekela HIV and Noncommunicable Disease Study and the Agincourt Health & socio-Demographic Surveillance System. Proportions are survey weighted

^a^Suppressed if viral load <400 copies/mL

^b^Unsuppressed if viral load ≥400 copies/mL

Preliminary mortality models indicated that the only control variables significantly associated with mortality were marital status and education. We therefore omitted the other control variables to maximize sample size (supplemental models with all controls, no controls, and alternate viral load thresholds showed similar results to our final models, see Additional Files 2-3).

**Fig. 2:**
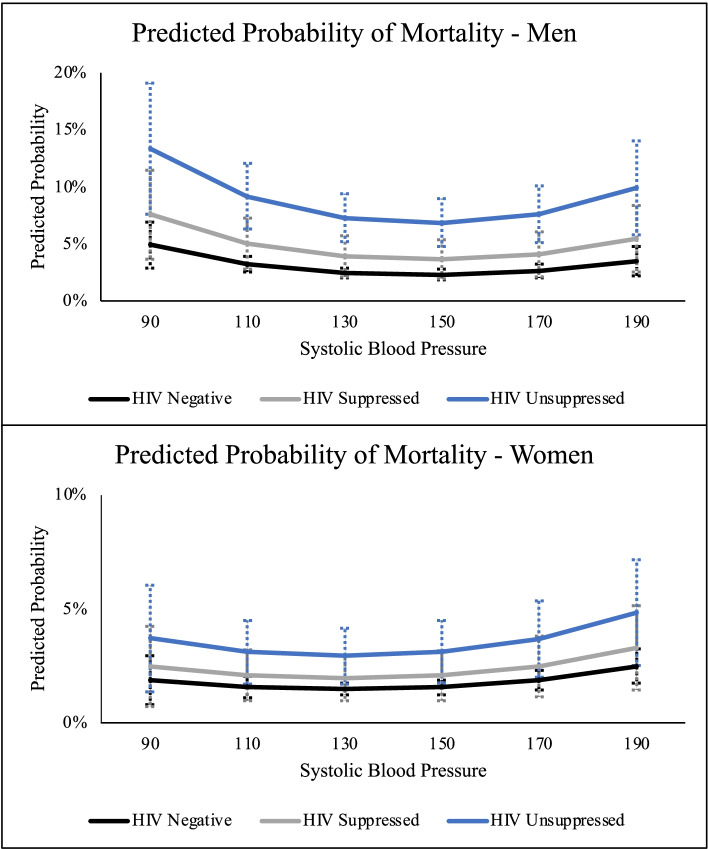
Predicted probability of mortality by sex, HIV status and systolic blood pressure, Agincourt, South Africa, 2010-2018

Table 2 presents the multivariate models. Results are similar for men and women. As expected, mortality increased with age and there was no significant additional mortality risk for PLWH with suppressed viral load. PLWH with unsuppressed viral load had higher odds of dying compared to those uninfected (men: adjusted odds ratio (aOR): 3.23, 95% CI: 2.21 to 4.71; women: aOR: 2.05, 95% CI: 1.27 to 3.30). There was a weak, non-linear relationship between systolic blood pressure and mortality (shown in Fig. 2). Adding an interaction between HIV status and systolic blood pressure did not significantly improve model fit (men: *p*=0.752; women: *p*=0.860). Men diagnosed with hypertension and currently on hypertension medication had 1.52 times the odds of dying compared to all other men (95% CI 1.05 to 2.21). Marriage and higher education were protective against mortality for men but showed no relationship for women.

**Table 2:** Multivariable logistic regression of all-cause mortality on HIV status, systolic blood pressure, and baseline characteristics, Agincourt, South Africa, 2010-2019

	**Men**^**d**^			**Women**^**e**^		
Covariates	**aOR**	**95% CI**	***P*-value**	**aOR**	**95% CI**	***P*-value**
Age	1.05	(1.04, 1.06)	<0.001	1.06	(1.04, 1.07)	<0.001
HIV Status [ref: HIV negative]						
HIV Positive Suppressed^a^	1.62	(0.97, 2.70)	0.06	1.34	(0.76, 2.34)	0.31
HIV Positive, Unsuppressed^b^	3.23	(2.21, 4.71)	<0.001	2.05	(1.27, 3.30)	0.00
Systolic Blood Pressure	0.93	(0.89, 0.97)	<0.001	0.96	(0.92, 1.01)	0.10
Systolic Blood Pressure Squared^c^	1.00	(1.00, 1.00)	<0.001	1.00	(1.00, 1.00)	0.05
Blood Pressure Medication	1.52	(1.05, 2.21)	0.03	1.32	(0.97, 1.80)	0.08
Marital Status [ref: single]						
Married/cohabiting	0.48	(0.32, 0.73)	<0.001	0.62	(0.35, 1.09)	0.10
Widowed/divorced	0.74	(0.46, 1.17)	0.20	0.95	(0.57, 1.59)	0.84
Education Level [ref: none/very low (≤3 years)]						
Primary (4-8 years)	0.69	(0.48, 0.97)	0.04	0.93	(0.61, 1.40)	0.72
Secondary school or higher (>8 years)	0.48	(0.28, 0.83)	0.01	0.85	(0.48, 1.52)	0.58
*N* 2,692	999			1,693		
Person Years	7,382			13,166		

^a^<400 copies/mL

^b^≥400 copies/mL

^c^Coefficients and 95% CI are small numbers which appear as 1.000 due to rounding

^d^Joint test of systolic blood pressure and systolic blood pressure squared (${\chi }_{2}^{2}=11.15;p=0.004)$

^e^Joint test of systolic blood pressure and systolic blood pressure squared (${\chi }_{2}^{2}=10.19;p=0.006)$

The overall strength of the relationship between unsuppressed viral load and mortality was much greater than the relationship between systolic blood pressure and mortality. For instance, men with unsuppressed viral load had about a 5% higher probability of dying compared to uninfected men (95% CI 0.029 to 0.074). In contrast, the probability of dying was about 1% higher for men with a systolic blood pressure of 170 mmHg compared to 140 mmHg (95% CI 0.002 to 0.018). In comparison, for women, there was a 1.6% higher probability of dying for women with unsuppressed viral load compared to uninfected women (95% CI 0.002 to 0.030) and about 0.4% higher probability for women with a systolic blood pressure of 170 mmHg compared to 140 mmHg (95% CI 0.0007 to 0.007).

## Discussion

Our study highlights the effectiveness of HIV treatment in a rural South African population in which both men and women had high HIV and hypertension prevalence. Over an eight-year follow-up period, mortality of those with suppressed viral load was not significantly different from those who were not living with HIV. Unsuppressed viral load, however, was associated with significantly increased risk of mortality. There was also a weak, non-linear concave relationship between systolic blood pressure and mortality risk.

Previous studies examining whether HIV infection and/or its treatment interact with blood pressure to increase mortality risk yielded mixed results [21–23]. In this study, we found no statistical interaction between HIV and systolic blood pressure in relation to mortality risk. However, our relatively small samples may have been underpowered to detect none but a large interaction.

A strength of the Ha Nakekela study is the use of a non-clinic, population-based sample including adults living with and without HIV. Our finding of elevated mortality for PLWH is important to situate in the local context. At the time of the baseline survey ART was just becoming widespread in the Agincourt HDSS study area [35, 36]. Our finding of elevated mortality risks for men and women with unsuppressed viral load aligns with evidence from the Africa Health Research Institute’s surveillance site (AHRI) in KwaZulu-Natal, which showed that mortality was increasingly concentrated in PLWH who had never sought care [37]. Continued mortality monitoring is needed to understand how this relationship may have changed over time, particularly as awareness and acceptance of HIV treatment became more widespread. Evidence from the ALPHA Network of demographic surveillance sites suggests that mortality among PLWH who are not on treatment is declining rapidly over time [38], which may be due in part to earlier diagnosis and treatment initiation so that untreated PLWH are increasingly people with early-stage disease.

Our finding of a non-linear relationship between systolic blood pressure and mortality aligns with results from a population-based cohort study at the national-level in South Africa, though they did not estimate separate models for men and women [39]. The authors suggested this weaker pattern compared to high-income countries may be due to competing mortality risks from HIV and TB predominately affecting ages 30-55 where systolic blood pressure is generally lower. Similarly, HIV/TB has also heavily impacted our study population, particularly in young adults [4]. Elevated mortality risks were associated with being on antihypertensive medication for men but not for women. It is possible that women are more likely to be engaged with the health care system [40], increasing their awareness and management of hypertension [10].

We found that the proportion of PLWH at baseline with a systolic blood pressure of 140mm Hg or more was lower compared to those who were not. This result accords with results from a longitudinal study in northwest South Africa that found that PLWH had a lower or similar blood pressure compared to those who were not living with HIV [41]. At the time of our study, under a third of men and just under half of women had suppressed viral loads. As ART has become more widespread, resulting survival gains, population ageing, and higher multimorbidity between HIV and cardiovascular risk factors may alter the patterns we observed [42, 43]. Further longitudinal follow-up with repeated measurements is needed in this population of older adults to assess the long-term cardiovascular effects of HIV and ART.

We acknowledge study strengths and limitations. We were able to include a long period of mortality follow-up. Our use of mortality data from population census updates also limits loss-to-follow-up, which has been shown to affect estimates from clinic-based follow-up studies [17]. Limitations include: that HIV status and systolic blood pressure were measured only at baseline; that despite high mortality, we lacked a sufficiently large sample size to investigate differential associations by cause of death; and that some respondents were excluded from the analysis due to missing data.

## Conclusions

Our results indicate that HIV and elevated blood pressure are acting as separate, non-interacting epidemics affecting high proportions of the older adult population. Despite relatively high levels of treatment, HIV continues to increase mortality for both men and women unless viral load is suppressed. Further, elevated blood pressure is increasing mortality for men and women. At present, the Agincourt HDSS is collecting data on COVID-19 in its surveillance site [44]. Future studies need to take into consideration the impacts of this third epidemic and its interactions with the advanced HIV and hypertension epidemics that continue to affect the lives of rural South Africans.

## Supplementary Information

**Additional file 1. **Multivariable logistic regression of all-cause mortality on HIV status, diastolic blood pressure and baseline characteristics, Agincourt, South Africa, 2010-2019.

**Additional file 2. **Multivariable logistic regression of all-cause mortality for men on baseline characteristics: no covariates, all covariates, and alternate viral load threshold on all-cause mortality, Agincourt, South Africa, 2010-2019.

**Additional file 3. **Multivariable logistic regression of all-cause mortality for women on baseline characteristics: no covariates, all covariates, and alternate viral load threshold, Agincourt, South Africa, 2010-2019.

## Data Availability

The datasets used and/or analysed during the current study are available from the corresponding author on reasonable request.

## Abbreviations

aOR: Adjusted odds ratio
ART: Antiretroviral therapy
HDSS: Health and socio-Demographic Surveillance System
NCD: Noncommunicable disease
PLWH: People living with HIV
