# Supplementary material for: Twin epidemics: the effects of HIV and systolic blood pressure on mortality risk in rural South Africa, 2010-2019
Source: BMC Public Health. 2022 Feb 24;22:387. doi: 10.1186/s12889-022-12791-z (PMC8866551; doi:10.1186/s12889-022-12791-z)
Supplement: Supplementary file 1 — Additional file 1. Multivariable logistic regression of all-cause mortality on HIV status, diastolic blood pressure and baseline characteristics, Agincourt, South Africa, 2010-2019. [file 12889_2022_12791_MOESM1_ESM.pdf]

Additional File 1. Multivariable logistic regression of all-cause mortality on HIV status, diastolic blood pressure and baseline characteristics, Agincourt, South Africa, 2010-2019.

	Men			Women		
Covariates	**aOR**	**95% CI**	**P-value**	**aOR**	**95% CI**	**P-value**
Age	1.049	(1.037, 1.062)	<0.001	1.060	(1.046, 1.074)	<0.001
HIV Status [ref: HIV negative]						
HIV Positive Suppressed[a]	1.564	(0.934, 2.619)	0.089	1.256	(0.710, 2.222)	0.434
HIV Positive, Unsuppressed[b]	3.237	(2.217, 4.726)	<0.001	1.966	(1.217, 3.175)	0.006
Diastolic Blood Pressure	0.918	(0.877, 0.961)	<0.001	0.926	(0.861, 0.995)	0.037
Diastolic Blood Pressure Squared[c]	1.000	(1.000, 1.000)	<0.001	1.000	(1.000, 1.000)	0.023
Blood Pressure Medication	1.618	(1.123, 2.330)	0.010	1.300	(0.953, 1.773)	0.098
Marital Status [ref: single]						
Married/cohabiting	0.483	(0.321, 0.726)	<0.001	0.602	(0.342, 1.058)	0.078
Widowed/divorced	0.710	(0.444, 1.137)	0.154	0.927	(0.554, 1.551)	0.773
Education Level [ref: none/very low (≤3 years)]						
Primary (4-8 years)	0.670	(0.470, 0.954)	0.026	0.937	(0.617, 1.424)	0.761
Secondary school or higher (>8 years)	0.454	(0.261, 0.792)	0.005	0.845	(0.472, 1.515)	0.572
N		1,000			1,697	
Person Years		7,388			13,193	

[a] <400 copies/mL.
[b] ≥400 copies/mL.
[c] Coefficients and 95% CI are small numbers which appear as 1.000 due to rounding.
