# Supplementary material for: Twin epidemics: the effects of HIV and systolic blood pressure on mortality risk in rural South Africa, 2010-2019
Source: BMC Public Health. 2022 Feb 24;22:387. doi: 10.1186/s12889-022-12791-z (PMC8866551; doi:10.1186/s12889-022-12791-z)
Supplement: Supplementary file 2 — Additional file 2. Multivariable logistic regression of all-cause mortality for men on baseline characteristics: no covariates, all covariates, and alternate viral load threshold on all-cause mortality, Agincourt, South Africa, 2010-2019. [file 12889_2022_12791_MOESM2_ESM.pdf]

Additional File 2. Multivariable logistic regression of all-cause mortality for men on baseline characteristics: no covariates, all covariates, and alternate viral load threshold on all-cause mortality, Agincourt, South Africa, 2010-2019.

	No Covariates			All Covariates			Alt. VL Threshold		
**Covariates**	**aOR**	**95% CI**	**P-value**	**aOR**	**95% CI**	**P-value**	**aOR**	**95% CI**	**P-value**
Age	1.050	(1.047, 1.067)	<0.001	1.049	(1.035, 1.063)	<0.001	1.050	(1.038, 1.062)	<0.001
HIV Status 1 [ref: HIV negative]									
HIV Positive Suppressed[a]	1.840	(1.114, 3.040)	0.017	1.498	(0.880, 2.550)	0.137			
HIV Positive, Unsuppressed[b]	3.437	(2.363, 4.998)	<0.001	3.406	(2.302, 5.040)	<0.001			
HIV Status 2 [ref: HIV negative]									
HIV Positive Suppressed[c]							1.595	(0.988, 2.575)	0.056
HIV Positive, Unsuppressed[d]							3.456	(2.345, 5.084)	<0.001
Systolic Blood Pressure	0.934	(0.852, 0.974)	0.002	0.932	(0.893, 0.972)	<0.001	0.930	(0.892, 0.970)	<0.001
Systolic Blood Pressure[e]	1.000	(1.000, 1.000)	0.002	1.000	(1.000, 1.000)	0.002	1.000	(1.000, 1.000)	<0.001
Blood Pressure Medication	1.389	(0.962, 2.005)	0.079	1.346	(0.921, 1.969)	0.125	1.517	(1.044, 2.204)	0.029
Marital Status [ref: single]									
Married/cohabiting				0.500	(0.323, 0.774)	0.002	0.490	(0.326, 0.737)	<0.001
Widowed/divorced				0.810	(0.498, 1.318)	0.396	0.770	(0.484, 1.226)	0.271
Education Level [ref: none/very low (<= 3 years)]									
Primary (4-8 years)				0.627	(0.431, 0911)	0.014	0.683	(0.481, 0.969)	0.033
Secondary school or higher (>8 years)				0.430	(0.247, 0.749)	0.003	0.473	(0.276, 0.811)	0.007

South African		1.250	(0.882, 1.773)	0.210	
Working		0.916	(0.629, 1.336)	0.650	
Income Tertile (ref: 1)					
2		1.108	(0.738, 1.665)	0.620	
3		1.290	(0.890, 1.869)	0.179	
N	999		943		999
Person Years	7,382		7,068		7,382

[a] <400 copies/mL.
[b] ≥400 copies/mL.
[c] <1,000 copies/mL.
[d] ≥1,000 copies/mL.
[e] Coefficients and 95% CI are small numbers which appear as 1.000 due to rounding.
