# Supplementary material for: Twin epidemics: the effects of HIV and systolic blood pressure on mortality risk in rural South Africa, 2010-2019
Source: BMC Public Health. 2022 Feb 24;22:387. doi: 10.1186/s12889-022-12791-z (PMC8866551; doi:10.1186/s12889-022-12791-z)
Supplement: Supplementary file 3 — Additional file 3. Multivariable logistic regression of all-cause mortality for women on baseline characteristics: no covariates, all covariates, and alternate viral load threshold, Agincourt, South Africa, 2010-2019. [file 12889_2022_12791_MOESM3_ESM.pdf]

Additional File 3. Multivariable logistic regression of all-cause mortality for women on baseline characteristics: no covariates, all covariates, and alternate viral load threshold, Agincourt, South Africa, 2010-2019.

	No Covariates			All Covariates			Alt. VL Threshold		
**Covariates**	**aOR**	**95% CI**	**P-value**	**aOR**	**95% CI**	**P-value**	**aOR**	**95% CI**	**P-value**
Age	1.064	(1.053, 1.076)	<0.001	1.059	(1.045, 1.074)	<0.001	1.058	(1.044, 1.071)	<0.001
HIV Status 1 [ref: HIV negative]									
HIV Positive Suppressed[a]	1.409	(0.819, 2.422)	0.215	1.341	(0.745, 2.412)	0.328			
HIV Positive, Unsuppressed[b]	2.165	(1.361, 3.442)	0.001	2.142	(1.324, 3.466)	0.002			
HIV Status 2 [ref: HIV negative]									
HIV Positive Suppressed[c]							1.302	(0.759, 2.232)	0.337
HIV Positive, Unsuppressed[d]							2.221	(1.361, 3.623)	<0.001
Systolic Blood Pressure	0.960	(0.916, 1.007)	0.091	0.953	(0.909, 0.999)	0.043	0.960	(0.916, 1.006)	0.088
Systolic Blood Pressure[e]	1.000	(1.000, 1.000)	0.046	1.000	(1.000, 1.000)	0.018	1.000	(1.000, 1.000)	0.043
Blood Pressure Medication	1.317	(0.968, 1.792)	0.08	1.325	(0.956, 1.837)	0.091	1.318	(0.968, 1.796)	0.080
Marital Status [ref: single]									
Married/cohabiting				0.631	(0.347, 1.148)	0.132	0.626	(0.356, 1.100)	0.104
Widowed/divorced				0.917	(0.527, 1.597)	0.760	0.957	(0.570, 1.601)	0.867
Education Level [ref: none/very low (<= 3 years)]									
Primary (4-8 years)				0.891	(0.575, 1.383)	0.607	0.929	(0.613, 1.408)	0.730
Secondary school or higher (>8 years)				0.839	(0.466, 1.513)	0.560	0.850	(0.477, 1.515)	0.582

South African				1.064	(0.751, 1.508)	0.725			
Working				1.153	(0.763, 1.743)	0.500			
Income Tertile (ref: 1)									
2				0.867	(0.581, 1.293)	0.484			
3				0.847	(0.595, 1.206)	0.356			
N		1,759			1,587			1,693	
Person Years		13,167			12,509			13,166	

[a] <400 copies/mL.
[b] ≥400 copies/mL.
[c] <1,000 copies/mL.
[d] ≥1,000 copies/mL.
[e] Coefficients and 95% CI are small numbers which appear as 1.000 due to rounding.
